# An Iterative and Targeted Sampling Design Informed by Habitat Suitability Models for Detecting Focal Plant Species over Extensive Areas

**DOI:** 10.1371/journal.pone.0101196

**Published:** 2014-07-14

**Authors:** Ophelia Wang, Luke J. Zachmann, Steven E. Sesnie, Aaryn D. Olsson, Brett G. Dickson

**Affiliations:** 1 Lab of Landscape Ecology and Conservation Biology, School of Earth Sciences and Environmental Sustainability, Northern Arizona University, Flagstaff, Arizona, United States of America; 2 Conservation Science Partners, Inc., Truckee, California, United States of America; 3 U.S. Fish and Wildlife Service, Albuquerque, New Mexico, United States of America; University of Saskatchewan, Canada

## Abstract

Prioritizing areas for management of non-native invasive plants is critical, as invasive plants can negatively impact plant community structure. Extensive and multi-jurisdictional inventories are essential to prioritize actions aimed at mitigating the impact of invasions and changes in disturbance regimes. However, previous work devoted little effort to devising sampling methods sufficient to assess the scope of multi-jurisdictional invasion over extensive areas. Here we describe a large-scale sampling design that used species occurrence data, habitat suitability models, and iterative and targeted sampling efforts to sample five species and satisfy two key management objectives: 1) detecting non-native invasive plants across previously unsampled gradients, and 2) characterizing the distribution of non-native invasive plants at landscape to regional scales. Habitat suitability models of five species were based on occurrence records and predictor variables derived from topography, precipitation, and remotely sensed data. We stratified and established field sampling locations according to predicted habitat suitability and phenological, substrate, and logistical constraints. Across previously unvisited areas, we detected at least one of our focal species on 77% of plots. In turn, we used detections from 2011 to improve habitat suitability models and sampling efforts in 2012, as well as additional spatial constraints to increase detections. These modifications resulted in a 96% detection rate at plots. The range of habitat suitability values that identified highly and less suitable habitats and their environmental conditions corresponded to field detections with mixed levels of agreement. Our study demonstrated that an iterative and targeted sampling framework can address sampling bias, reduce time costs, and increase detections. Other studies can extend the sampling framework to develop methods in other ecosystems to provide detection data. The sampling methods implemented here provide a meaningful tool when understanding the potential distribution and habitat of species over multi-jurisdictional and extensive areas is needed for achieving management objectives.

## Introduction

As a leading threat to global biodiversity, non-native plant invasions can reduce species richness and facilitate changes in ecosystem structure and functioning [1]–[3]. In arid and semi-arid ecosystems, the positive interaction between annual and perennial invasive grass cover, increased loading of fine-fuels, burning frequency, and fire severity illustrates the potential for plant invasion to substantially alter disturbance patterns, especially regional fire regimes [4], [5]. Increases in fire frequency, size, and intensity facilitated by invasive species can promote ongoing invasion while populations of non-fire adapted native plants are slow to recover or show a decline [5], [6]. Because disturbances in arid ecosystems involve slow vegetation recovery and a loss of native biodiversity [7], [8], targeted and adaptive management activities are critical in order to mitigate the negative impacts of non-native invasive plants.

Ideally, adaptive management entails determining invasion risk as well as prioritizing management actions to prevent new introductions to suitable but uncolonized habitats. Precisely determining the occurrence of non-native invasive plants is essential for robust prioritization and mitigation efforts [9]. These data can, in turn, be used to develop and refine probability of occurrence (i.e. “early-warning”) maps that help to target control and prevention activities. Most land management agencies, however, do not have the capacity to survey more than 1–2% of land within their ownerships [10]. Therefore, a multi-jurisdictional sampling approach can play a critical role in integrating resources and evaluating the extent of plant invasions. This type of sampling can accommodate management needs and accomplish multiple survey objectives, such as detecting invasions in early stages, locating populations of multiple invasive species, or detecting large and problematic populations from a fire and fuels management perspective [9], [10].

Invasive plant sampling frequently takes place opportunistically based on expert knowledge, where investigators explore areas known to have infestations, or along roadsides and in residential areas in semi-regular increments of distance along transportation corridors [11]–[13]. Although rapid and opportunistic sampling can inform coarse-level species distribution, this approach will typically incorporate non-detection sampling bias (i.e. species may be present but undetected) [14], [15], especially for species that can occur away from transportation routes. Additionally, because sampling in areas known to be invaded may not reflect the species' true realized niche [16], a more suitable design should sample across environmental gradients to capture conditions that influence species distributions by leveraging existing plant occurrence records and knowledge regarding potential habitat characteristics and plant phenology [17], [18].

Statistically based habitat suitability models (HSMs) use empirical relationships between species occurrence and environmental factors to predict habitat suitability across the landscape [19]–[21]. Sampling across wide ranges of predicted habitat suitability can facilitate characterization of the environmental attributes of locations where a species can potentially establish [22]. A HSM-informed sampling approach is also feasible for increasing species detectability by identifying locations of relatively high habitat suitability [23], [24]. It can help focus search efforts to locations of suitable but previously undetected habitats to refine understanding of the current extent of invasions and reduce time and transportation costs by avoiding areas of extremely low habitat suitability. Furthermore, iterative HSM-informed sampling efforts can improve existing HSMs with new field data to better characterize the distribution of invasive species [25]. A study that employed this approach with targeted sampling efforts showed better model performance and greater species detection than non-targeted sampling [26]. Drawing from previous work, we explored a generalizable iterative process of using HSMs (fit using ancillary geospatial data) to guide initial targeted sampling efforts, integrating new data to improve HSMs, and compiling refined models to help direct more rigorous future sampling efforts, as well as improve sampling efficiency and detection rates.

Here we present a HSM-informed and targeted sampling design to gain efficiencies in sampling and improve detection rates over extensive areas and multiple land management jurisdictions. For five non-native invasive plant species in the Sonoran Desert region of the southwestern U.S., our specific objectives were to: 1) use existing non-native invasive plant data to model relationships between environmental characteristics and the occurrence of each species; 2) identify highly suitable habitats and areas of previously undetected but with potential for invasion by each species using HSMs; 3) improve knowledge of the range of environmental conditions occupied by each species by simultaneously sampling in low-medium suitability areas; and 4) develop an iterative and targeted sampling framework by coupling existing and newly collected data to improve HSMs and detections of invasive species in the field. Our design aimed to satisfy the pressing need of land managers to detect previously unknown non-native invasive plant populations and characterize their distribution over extensive and less accessible areas.

## Materials and Methods

### Study area and focal species

**Figure 1:**
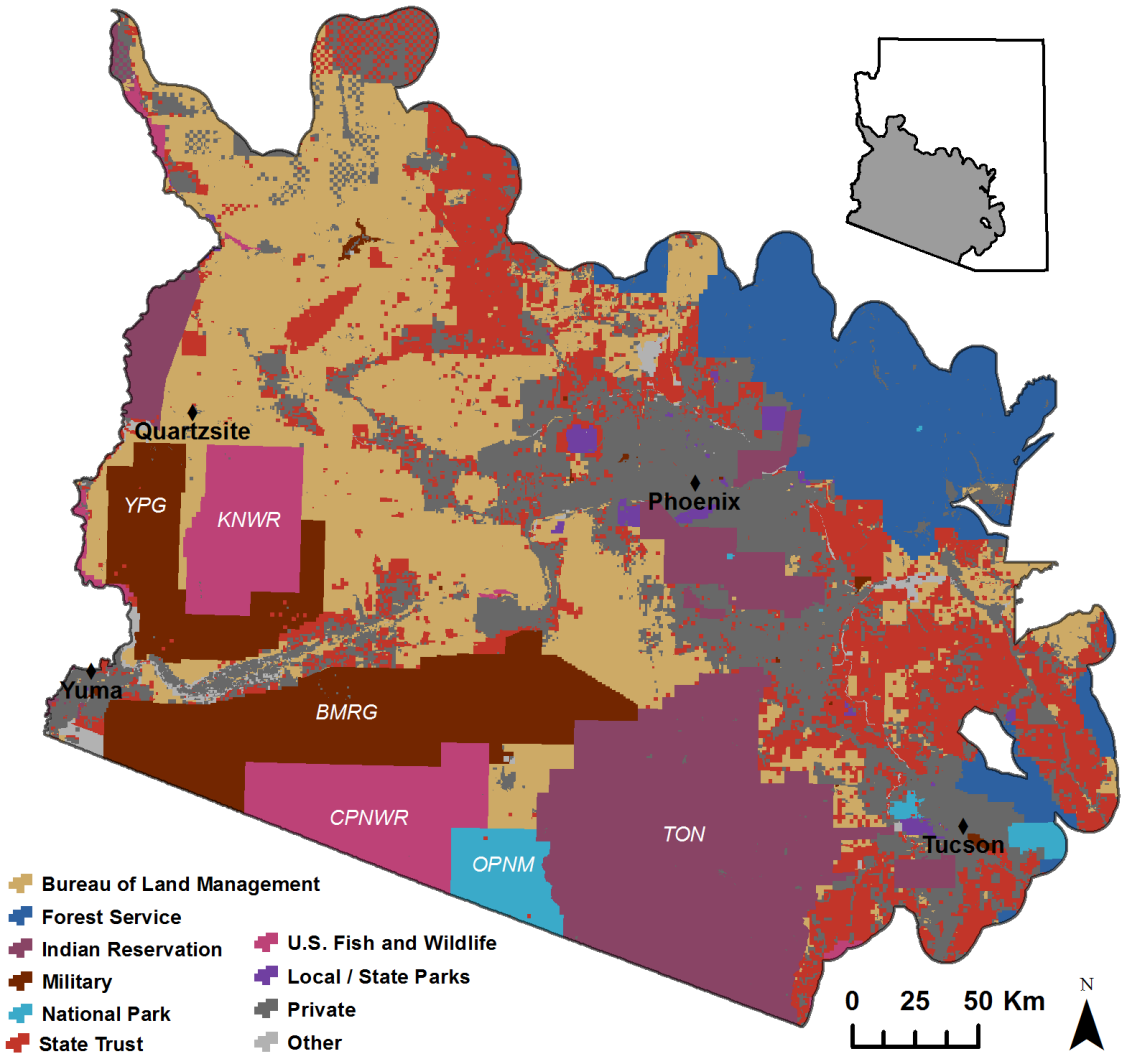
Boundary and land jurisdictions of our study area in the Sonoran Desert of Arizona. Specific land ownerships highlighted by abbreviations and include: the U.S. Army Yuma Proving Ground (YPG), Barry M. Goldwater Air Force Range (BMGR), Kofa National Wildlife Refuge (KNWR), Cabeza Prieta National Wildlife Refuge (CPNWR), Organ Pipe Cactus National Monument (OPCNM), and the Tohono O'odham Nation (TON).

Our study area in the Sonoran Desert of Arizona encompassed multiple land ownerships, including lands administered by the U.S. Army Yuma Proving Ground (YPG; authorized by L. Merrill), Barry M. Goldwater Air Force Range (BMGR) East (authorized by R. Whittle and T. Walker), BMGR West (authorized by A. Rosenberg), Bureau of Land Management (BLM; authorized by E. Faulkner), Kofa National Wildlife Refuge (KNWR; authorized by S. Henry), Cabeza Prieta National Wildlife Refuge (CPNWR; authorized by S. Barclay), Organ Pipe Cactus National Monument (OPCNM; authorized by S. Rutman), Tohono O'odham Nation (TON; authorized by K. Howe), Saguaro National Park (authorized by D. Backer), Sonoran Desert National Monument (authorized by R. Hansen), and Ironwood National Monument (authorized by D. Tersey) (Figure 1). The total area available for field sampling was 66,541 km^2^ after excluding inaccessible areas in private properties, state trust lands, and a small number of Native American lands. The study area included Arizona Upland and Lower Colorado River Valley subdivisions of the Sonoran Desert vegetation [27], as well as extensive areas dominated by native and non-native invasive plant species recently impacted by large-scale fire events. Most of the low-lying desert ecosystems in this region had received extremely low annual rainfall. Notably, long-term (1952–2007) average annual precipitation at the YPG and KNWR was 93 mm and 175 mm, respectively (i.e. the military and the U.S. Fish and Wildlife Service lands between Yuma and Quartzsite shown in Figure 1) (http://www.prism.oregonstate.edu/). The study area also encompassed considerable topographic relief resulting from mountain ranges separated by expansive desert valleys, plains, and broad alluvial aprons (bajadas), with an elevation range from 25 m in the western lowlands to approximately 1,500 m in the KNWR.

We focused on five non-native invasive plants identified as important or of current and future management concern by scientists and regional land managers within our study area, including two annual C3 grasses: red brome (*Bromus madritensis* var. *rubens*; hereafter referred to as *Bromus*) [28], [29] and Mediterranean grass (including *Schismus arabicus* and *S. barbatus* that are indistinguishable in the field and have been assessed as one plant group by desert botanists [30]; hereafter referred to as *Schismus*) [31], [32]; two annual cruciferous forbs: Sahara mustard (*Brassica tournefortii*; hereafter referred to as *Brassica*) [33], [34] and arugula (*Eruca vesicaria* ssp. *sativa*; hereafter referred to as *Eruca*); and one perennial C4 grass: buffelgrass (*Pennisetum ciliare*; hereafter referred to as *Pennisetum*) [35], [36]. Spring wildfires in the Sonoran Desert occurring in the last several decades have shown association with positive El Niño Southern Oscillation events [37], [38]. In particular, *Pennisetum* is reliant on summer monsoonal precipitation and is recognized as a potential threat in this ecosystem because of its drought hardiness and tendency to accumulate hazardous levels of flammable biomass throughout the dry season [39], [40]. *Bromus*, *Schismus*, and *Brassica* can potentially create a continuous fine fuel loads in areas where fuels are spatially scarce and increase fire return intervals [28], [31], [34].

### Development of habitat suitability models

We compiled known locations of non-native invasive plants from published and unpublished databases, including the Southwest Environmental Information Network (SEINET; http://swbiodiversity.org/seinet/index.php), the Southwest Exotic Mapping Program (SWEMP; http://sbsc.wr.usgs.gov/research/projects/swepic/swemp/swempA.asp), multi-year invasive survey data from managers of the BLM and National Park Service, and unpublished research data from local and regional biologists. Using these data, we retained only geographic locations (*n* = 9,713; 2,783 for *Bromus*, 615 for *Schismus*, 1,476 for *Brassica*, 95 for *Eruca*, and 4,744 for *Pennisetum*) at which plant density of >2 individuals per hectare was documented to address potential errors introduced by the uncertainty of species occurrence information.

We used environmental variables that reflected principal biophysical characteristics of the study area that were previously defined as important for habitat suitability modeling of non-native invasive plants, including topographic [39], precipitation [41], spectral [42], and road variables [43]. We obtained the environmental data from the National Elevation Dataset (NED; http://ned.usgs.gov/), terrain and radiometrically calibrated Landsat Thematic Mapper (TM) imagery from August 2009 (U.S. Geological Survey EROS Data Center; http://edc.usgs.gov), precipitation from the Parameter-elevation Regressions on Independent Slopes Model (PRISM; http://www.prism.oregonstate.edu/), and rasterized road data derived from the 2003 TeleAtlas Dynamap Transportation version 5.2 product (Spatial Insights, Inc.) (Table 1). For topographic variables, we smoothed the digital elevation model to reduce visually discernible contour and point artifacts and derived slope and aspect variables (sine- and cosine-transformed to represent slope eastness and northness, respectively). To capture precipitation patterns, we summarized winter (December-March) and summer (June-September) months and derived mean annual, winter, and summer precipitation layers from 2000–2009 using the PRISM data. To characterize soil substrate types, we used the continuous spectral information obtained from six TM bands (bands 1–5 and 7) of eight Landsat image scenes (path/row p36/r37, p36/r38, p37/r36, p37/r37, p37/r38, p38/r36, p38/r37, and p38/r38) from August 2009. The rationale was that spectral characteristics of soil substrates of high sand content or loose texture soils appeared to be highly related to the presence of three focal species (*Brassica*, *Schismus*, and *Eruca*). We converted the digital numbers of these radiometrically corrected TM images into spectral reflectance values and then mosaicked images by using ENVI version 4.7.1 (ITT Visual Information Solutions, Inc.). We also generated the summer Normalized Difference Vegetation Index (NDVI) using reflectance information of TM red and near-infrared (NIR) bands (NIR-Red/NIR+Red) to represent patterns of vegetation greenness. To quantify road proximity, we calculated the Euclidian distance from a raster cell to the nearest road. We obtained or derived all variables at a 30-m pixel resolution using ArcGIS version 10 (Esri, Inc.).

**Table 1:** List of environmental variables used in habitat suitability models at cell size  = 30 m for stratifying our sampling locations in the Sonoran Desert of Arizona in the 2011 field season.

Variable type	Variable
Topography	Elevation
	Slope
	Aspect (eastness)
	Aspect (northness)
Spectral (August 2009)	TM band 1
	TM band 2
	TM band 3
	TM band 4
	TM band 5
	TM band 7
	NDVI
Precipitation (2000–2009)	Mean annual
	Mean summer (7–81 mm)
	Mean winter (10–103 mm)
Road proximity	Euclidean distance to the nearest road

TM  =  Landsat Thematic Mapper imagery; NDVI  =  Normalized Difference Vegetation Index.

We developed five separate HSMs for each species (total  = 25 models) using a maximum entropy algorithm and the Maxent software package version 3.3.3e (http://www.cs.princeton.edu/~schapire/maxent/) [44], [45]. For HSMs that rely solely on presence-only data, environmental conditions are typically represented by occurrence records and background data randomly drawn from the entire region, whereas species occurrence data tend to be spatially biased toward locations with easy access. To account for such bias, Phillips et al. proposed to select background sample locations with the same sampling bias as species presence records [46]. We employed a “bias prior” approach in our background data based on the density of sampled locations of all focal species across our study area and an estimate of relative sampling effort as recommended by Merow et al. [47]. We assigned raster cell value  = 1 for cells with presence records of all focal species to represent sampling intensity and a “no data” value for the remaining cells [48]. For each focal species we constructed five separate HSMs that each combined environmental variables as follows: Model 1) topography, spectral bands, NDVI, and precipitation data; Model 2) topography, spectral bands, NDVI, precipitation layers, and road distance; Model 3) topography, spectral bands, NDVI, and road distance; Model 4) topography, spectral bands, NDVI, and winter or summer (for *Pennisetum*) precipitation; and Model 5) topography, spectral bands, NDVI, winter or summer (for *Pennisetum*) precipitation, and road distance (Table 1).

Each model included a bias estimate and employed the hinge algorithm (i.e. piece-wise linear regression) to develop HSMs with ten replicates at the convergence threshold of 10^−5^ (i.e. where model training terminated in terms of log loss per iteration). We used 60% of the occurrence data for model training and the remaining 40% for testing [44], [49]. We evaluated the contribution for each variable by randomly permuting the values of that variable among the presence and background training points and measuring the resulting decrease in training area under the receiver operating characteristic curve (AUC) [50]. A large decrease indicated a strong dependence on that particular variable. We also evaluated variable importance by omitting each variable in turn and then using it in isolation [50]. The results based on training and test gain informed how the variable, when omitted or used alone, affected model gain. The result based on AUC informed how the variable influenced the model in predicting presences in the data.

Model performance evaluation took place using three threshold independent assessment measures to avoid using arbitrary binary threshold presence/absence when the assumption for the threshold could not be validated. We first constructed null models using randomly created sampling points to confirm that all our HSMs for each species had significantly higher values of training AUC than random models (α = 0.05) [51]. We then used the AUC values of >0.70 to determine acceptable model performance [52], [53]. We also calculated the point biserial correlation (COR) as Pearson's correlation coefficient *r* between predicted suitability and presence/pseudo-absence of the test data to examine how well calibrated the predicted suitability was in correspondence to the probability of presence of each focal species. (α = 0.05) [49].

### Sampling location stratification and selection in 2011

Stratified random and targeted sampling has been a well-recognized approach for estimating landscape-level infestation and characterizing invasion [10], [26], [54] Previous stratification studies applied ensemble forecasting to combine multiple model outputs into a single projection for reducing individual model errors [24], [55], [56]. However in our study, locations with the highest habitat suitability (i.e. 90^th^ percentile) that were completely overlapped by all five HSMs for each species only covered <5% of the study area, making these areas less representative of habitat conditions across the region. Therefore, we combined multiple models described above and stratified the highly suitable habitats suggested by at least one of the five HSMs for each species to identify potential sampling locations. We randomly selected field sampling locations within areas of the 90^th^ percentile of separate HSMs for each focal species but confined them within areas of low slopes and proximity to improved paved and unimproved dirt roads in more remote locations. The rationale for selecting areas based on slope (≤20 degrees) and proximity to roads (250 m-2 km) was to reduce the amount of effort required to access field locations and increase sample size. Additionally, most of our focal species prefer soil conditions on low slopes to area of steep rocky terrain. The road proximity threshold was based on the influence of roads (e.g. enhanced moisture, fertilization, and dispersal of invasives) which could extend from an unimproved road or major highway in the Sonoran Desert [32]. We included all access roads visible in acquired data layers for sampling, such as rugged four-wheel drive and off-highway vehicle roads with access to backcountry locations. Next, we implemented a spatially balanced approach to identify potential locations with a weighted representation of suitable habitats based on each HSM for each species across available sampling areas. The potential sample locations were well distributed across remote portions of the study area. The approach was based on using specific raster cells values (i.e. weights of habitat suitability) to determine the inclusion probability of a location to be sampled [57], [58].

Our focal species *Brassica*, *Schismus*, and *Eruca* favor disturbed or loose sandy soils in the study area [32], [43]. To further prioritize sample locations suited for these species, we discriminated sandy soils from other soil substrates such as basalt and desert pavement using TM imagery. We employed linear spectral unmixing (e.g. [59]) to estimate the proportion of sand substrate within a pixel and then applied a pixel growing technique (e.g. [60]) to extract adjacent pixels within two standard deviations of the mean value of seed pixels of pure sand. We then used pixel values from the unmixing step to represent the proportion of a pixel dominated by sand (where 0 =  no sand and 1 = 100% sand) for assigning five very low to high sandiness categories. We overlaid the sandiness category layer with a buffer range placed around the center pixel of a potential location to assign the sandiness based on a majority count of pixels. We directed our crews to allocate a greater sampling effort, when logistically feasible, to reach accessible plot locations that occurred on sites of high to medium sandiness.

### Field data collection

**Figure 2:**
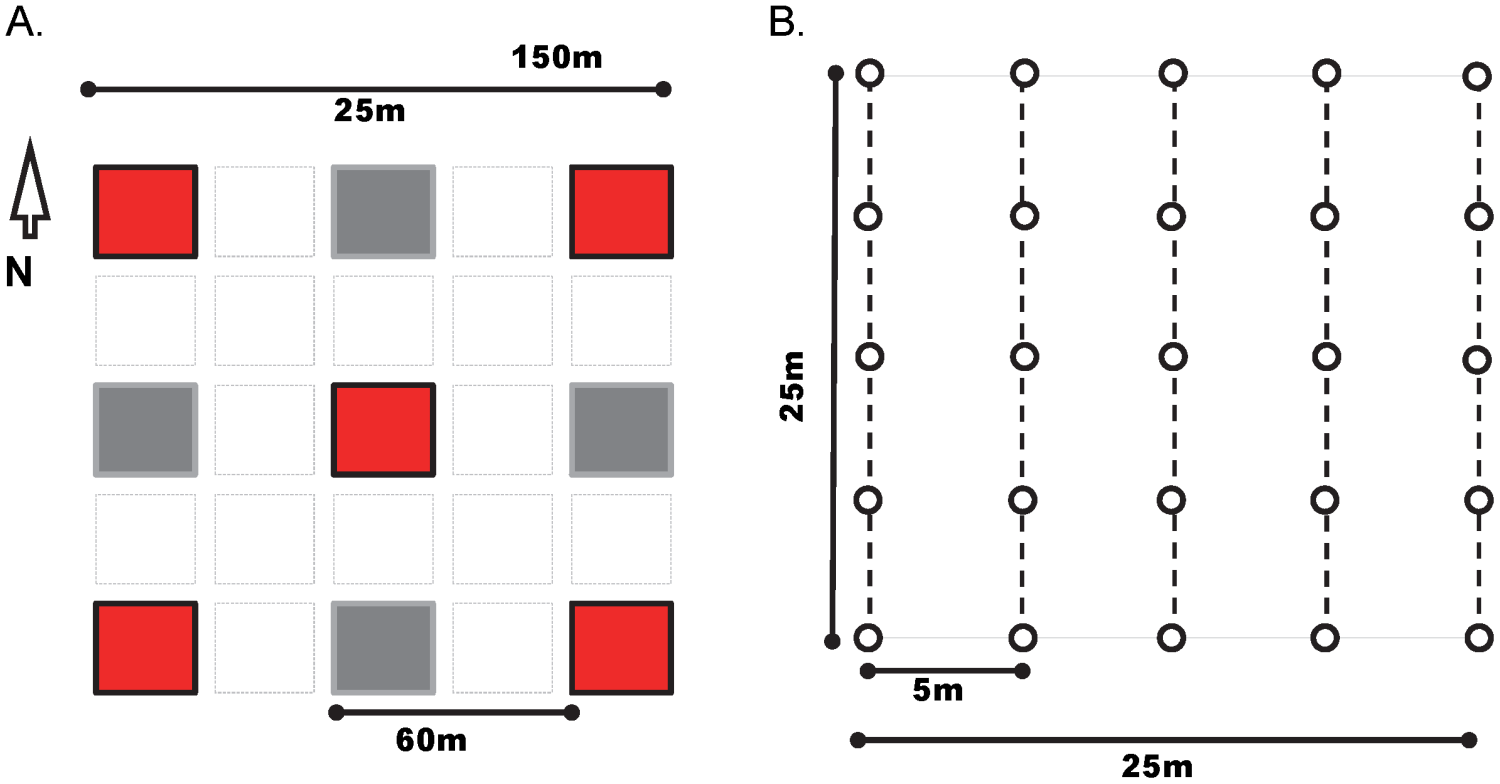
Nested pixel plot design used to sample plants in the Sonoran Desert of Arizona. A) Plot were co-registered with the resolution and location of a MODIS image pixel, and included five nested subplots, each co-registered with the resolution and location of a Landsat TM image pixel. Target and alternate (used when the target subplot was inaccessible) subplots are in red and gray, respectively. B) Within each subplot, five point-intercept transects were established to measure attributes of species composition at 5 m intervals.

We collected field data from 238 plots between late January and April of 2011, the principal growing season for most annual and perennial herbaceous plants in the study landscape. To allow our field data to match the spatial resolution of two differing remote sensing platforms used for occurrence modeling (Olsson et al., *Ecological Modelling*, in review), we adopted a nested plot design to enable the approximate alignment between sampled locations and satellite image pixels [61]. We spatially geo-registered each plot with a Moderate Resolution Imaging Spectroradiometer (MODIS) image pixel (250×250 m) and five nested subplots each with a Landsat TM image pixel (30×30 m; Figure 2A). The rationale was to precisely match field data with the pixel location and resolution of both sensor types (i.e. MODIS and TM) used for developing time-series and phenology-based models of non-native invasive plant occurrence (Olsson et al., *Ecological Modelling*, in review). Geographically co-registered and multi-scaled plots enabled our capacity to reduce error introduced by mismatches of scale and location between field and remote sensing data [62]. Crews used the geographic coordinates of the pixel corner of subplots and then navigated to the corner using a Magellan MobileMapper 6 Global Positioning System (GPS) receiver. Within each subplot, crews established 25 point intercepts where a pin flag intersected a transect line, along five transects at every five meters (Figure 2B). We recorded species name and substrate at each point intercept for both native and non-native invasive plants, as well as presence/absence of our focal species and disturbance types within each subplot.

### Relating habitat suitability models to field detections in 2011

To assess how well predicted habitat suitability corresponded to detections of focal species in 2011, we used a generalized linear model (GLM) to examine the relationship between detection (i.e. binary presence/absence outcome for each focal species at each subplot) and habitat suitability predicted by each HSM. We then modeled the detection of each focal species with a binomial distribution and a logit link function in the R statistical package version 3.0.2 (http://www.r-project.org). The intent of this analysis was to provide an indication of how well the HSM-informed stratification directed us to sampling locations where species detections were more likely. To assess model fit, we calculated the difference in values of Akaike's Information Criterion (ΔAIC; [63]) between a detection model that included predicted habitat suitability and an intercept-only model. We considered suitability models with ΔAIC >10 as good approximations of the data [63].

### Next iteration of targeted sampling in 2012

Based on our sampling efforts from 2011, we developed a more rigorous and targeted sampling strategy for a 2012 (late February-April) field season in order to increase detection rates and sample size. This targeted strategy is extendible to other species and ecosystems. We collected data from 506 plots by integrating a more targeted design of: 1) adding the 2011 detection data into species occurrence records to develop improved HSMs; 2) using a greater range of habitat suitability (i.e. 70^th^ percentile) to cover more local habitat variation to stratify potential sampling locations; 3) also stratifying sampling efforts to areas to where the ratio of maximum spring NDVI to mean spring NDVI of MODIS imagery between 2001 and 2010 exceeded the 60^th^ percentile to highlight locations with annual plant production higher than average greenness and reflect strong herbaceous growth during a wet growing season; 4) further allocating locations to areas with greater than average MODIS NDVI acquired in early 2012 to focus sampling efforts on areas that had received sufficient precipitation in winter 2011/spring 2012 for seed germination and seedling growth; and 5) identifying stratified random, spatially balanced locations and then constraining these potential locations to areas containing 4–5 plots within 450–650 m of one another [20]. Crews aimed to sample an average of five locations per day in these areas and maintain a minimum travel distance between daily visits of 10–20 km. Previous work demonstrated that despite potential bias introduced by sampling at locations within relatively close distance, certain statistical estimators could provide unbiased estimates of abundance and density for species with low abundance in local populations [64], [65]. The nested pixel plot design at each plot and transect-based point intercept data collection at each subplot remained the same as field samples collected in 2011.

## Results

### Environmental attributes of sampled locations in 2011

In 2011, our five 2-person field crews sampled 238 plots and 1,171 subplots (a small number of subplots were inaccessible and did not equal five per plot). All subplot locations selected for sampling had an average GPS error of 3.6 m (standard deviation  = 10.3 m) by calculating distance between a GPS-recorded subplot corner and a location of the corresponding subplot corner assigned to crews. We sampled 157 locations (66%) on BLM lands, 35 (14.7%) on Native American reservations, 21 (8.8%) on military lands, and 25 (10.5%) on U.S. Forest Service lands, national wildlife refuges, and state and national parks.

Eleven vegetation types classified by the Landfire Existing Vegetation Type 1.1.0 data (http://www.Landfire.gov/NationalProductDescriptions21.php) appeared among our 2011 sampled locations, including the dominant Sonoran palo-verde-mixed cacti desert scrub, Sonora-Mojave creosote bush-white bursage desert scrub, and North American warm desert riparian and sparsely vegetated systems. Many locations were in areas highly disturbed by animal burrowing and grazing (38% of all), vehicular and human traffic infrastructure (18%), and fire and erosion (17%). We detected soil disturbance in 71% of sampled locations, which appeared to be associated with anthropogenic factors, since our sampling locations were ≤2 km of roads and were exposed to past or ongoing disturbances.

### Characteristics of habitat suitability models and model predictions

We found that Model 4 for the winter annuals and Model 5 for *Pennisetum* predicted highly suitable habitats that were also well-known areas with great focal species abundance. For example, the environmental attributes of highly suitable habitats (i.e. 90^th^ percentile) for *Pennisetum* reflected known habitat conditions in elevation, slope, aspect, annual precipitation, and vegetation type. These models reflected the known importance of seasonal precipitation events for our five focal species to green up (e.g. initiating germination and seedling growth), as well as the known influence of road proximity on the dispersal and colonization of *Pennisetum*. Overall, elevation, winter or summer precipitation, and slope appeared to be the most important variables for our focal species HSMs. The most important variable was elevation for *Brassica*, *Schismus*, and *Eruca*, winter precipitation for *Bromus*, and summer precipitation for *Pennisetum*. The second most important variable was slope for *Eruca* and winter precipitation for *Schismus*. Elevation accounted for 65%, 51%, and 38% of model contribution for Model 4 for *Brassica*, *Schismus*, and *Eruca*, respectively. Winter precipitation showed 34% and 21%, respectively, of model contribution to Model 4 for *Bromus* and *Schismus*. Slope accounted for 31% of model contribution to Model 4 for *Eruca*. For Model 5 for *Pennisetum*, 56% of model contribution came from summer precipitation. Likewise, evaluation of variable importance showed that the most important variables, when used in isolation or when omitted, were elevation for *Brassica*, *Schismus*, and *Eruca*, winter precipitation for *Bromus*, and summer precipitation for *Pennisetum*.

All 25 HSMs outperformed null models constructed with random sampling points, showing AUC values that were significantly higher and deviated from what would be expected by random chance (*p*<0.05). AUC values from training and test data of all 25 HSMs indicated satisfactory model performance, with all values >0.70 (0.73–0.97 for training AUC and 0.71–0.93 for test AUC; Table 2). Species presence/pseudo-absence was significantly correlated with predicted habitat suitability of all 25 HSMs with notable variation among species (0.36–0.8 for COR, *p*<0.01 or <0.0001). Overall, COR values were the highest for *Eruca* and *Brassica* and lowest for *Bromus*, reflecting different degrees of dispersion and variability in suitability predicted at locations where species presence was recorded (Table 2).

**Table 2:** Average training and test receiver operating characteristic curve (AUC) and average point biserial correlation (COR) (±95% confidence interval) among the ten replicates for each focal species habitat suitability model used for our 2011 sampling location stratification in the Sonoran Desert of Arizona.

Species	Model (n = 10)	Training AUC	Test AUC	COR (Pearson's *r*)
*Schismus* (615)	1	0.86±0.01	0.81±0.02	0.51±0.09**
	2	0.87±0.01	0.79±0.02	0.45±0.05**
	3	0.85±0.01	0.78±0.01	0.61±0.08***
	4	0.84±0.01	0.78±0.02	0.54±0.05***
	5	0.84±0.01	0.79±0.01	0.57±0.06***
*Brassica* (1,476)	1	0.84±0.004	0.8±0.01	0.66±0.06***
	2	0.84±0.01	0.8±0.01	0.66±0.11***
	3	0.83±0.01	0.8±0.01	0.67±0.02***
	4	0.84±0.01	0.8±0.01	0.6±0.07***
	5	0.83±0.004	0.8±0.01	0.54±0.11**
*Bromus* (2,783)	1	0.75±0.01	0.73±0.01	0.4±0.005***
	2	0.76±0.01	0.74±0.01	0.46±0.05***
	3	0.73±0.01	0.71±0.01	0.35±0.05**
	4	0.75±0.01	0.72±0.01	0.37±0.04**
	5	0.75±0.01	0.72±0.01	0.36±0.04**
*Eruca* (95)	1	0.96±0.01	0.91±0.02	0.8±0.005***
	2	0.97±0.01	0.93±0.02	0.75±0.04***
	3	0.96±0.01	0.91±0.02	0.74±0.03***
	4	0.97±0.01	0.92±0.02	0.77±0.01***
	5	0.96±0.01	0.9±0.01	0.75±0.06***
*Pennisetum* (4,744)	1	0.78±0.003	0.76±0.004	0.56±0.05***
	2	0.78±0.003	0.76±0.004	0.59±0.06***
	3	0.75±0.01	0.74±0.01	0.48±0.01***
	4	0.77±0.002	0.75±0.01	0.53±0.05***
	5	0.76±0.003	0.75±0.004	0.57±0.06***

Number in parenthesis after each species  =  number of occurrence records in the Maxent model input. Model numbers referred to variables that included: 1) topography, spectral bands, NDVI, and precipitation data; 2) topography, spectral bands, NDVI, precipitation layers, and road distance; 3) topography, spectral bands, NDVI, and road distance; 4) topography, spectral bands, NDVI, and winter or summer (for *Pennisetum*) precipitation; and 5) topography, spectral bands, NDVI, winter or summer (for *Pennisetum*) precipitation, and road distance. **  = *p*<0.01, ***  = *p*<0.0001.

Model 4 for the winter annuals and Model 5 for *Pennisetum* together showed that 81% of the study area was within high predicted habitat suitability (i.e. 70^th^ percentile) for at least one of the five species. In particular, 38%, 29%, and 12% of the study area corresponded to the 70^th^ percentile of suitable habitats for one, two, and three focal species, respectively. Only 19.3% of the study area was not within high suitability for any given species. Areas with low to medium suitability appeared to be widely distributed. We found inter-model variability for all five focal species in areas predicted with high suitability. For example, 44% and 38% of the study area was respectively within the 70^th^ percentile predicted by at least one of the five HSMs for *Schismus* and *Brassica*, but all five *Schismus* and *Brassica* HSMs only completely overlapped in 18% and 21% of the study area, respectively. The variability was the lowest for *Eruca*, showing 33% of the study area covered by at least one HSM and 20% completely overlapped by all five HSMs.

All 25 HSMs, when overlapped together, predicted 39% of the study area with low (i.e. 30^th^ percentile) to very high habitat suitability for the five focal species with notable variation among species. We found that suitable habitats for *Schismus*, *Brassica*, and *Bromus* were predicted to be widespread, whereas *Eruca* and *Pennisetum* were less common across the study area. All five HSMs for each species predicted 59%, 64%, and 55% of the study area with low to very high suitability for *Brassica*, *Schismus*, and *Bromus*, respectively. In contrast, all five HSMs predicted 42% and 58% of the study area to have very low suitability (i.e. below 30^th^ percentile) for *Eruca* and *Pennisetum*, respectively.

### Sampled locations in 2011 across habitat suitability ranges

We sampled on locations predicted to harbor very high habitat suitability for one focal species, but low to medium suitability for other species to capture the range of environmental conditions occupied by each species. For instance, 28% and 62% of the sampled locations were respectively within the 90^th^ and 70^th^ percentile of *Brassica* habitat suitability, and were also within areas of lower suitability for at least one of the other four species. Likewise, 16% and 39% of the total locations that were within the 90^th^ and 70^th^ percentile suitability for *Schismus*, respectively, fell within lower habitat suitability of at least one of the other four species. Fewer sampled locations were within areas of high habitat suitability for *Bromus* and *Pennisetum*.

**Figure 3:**
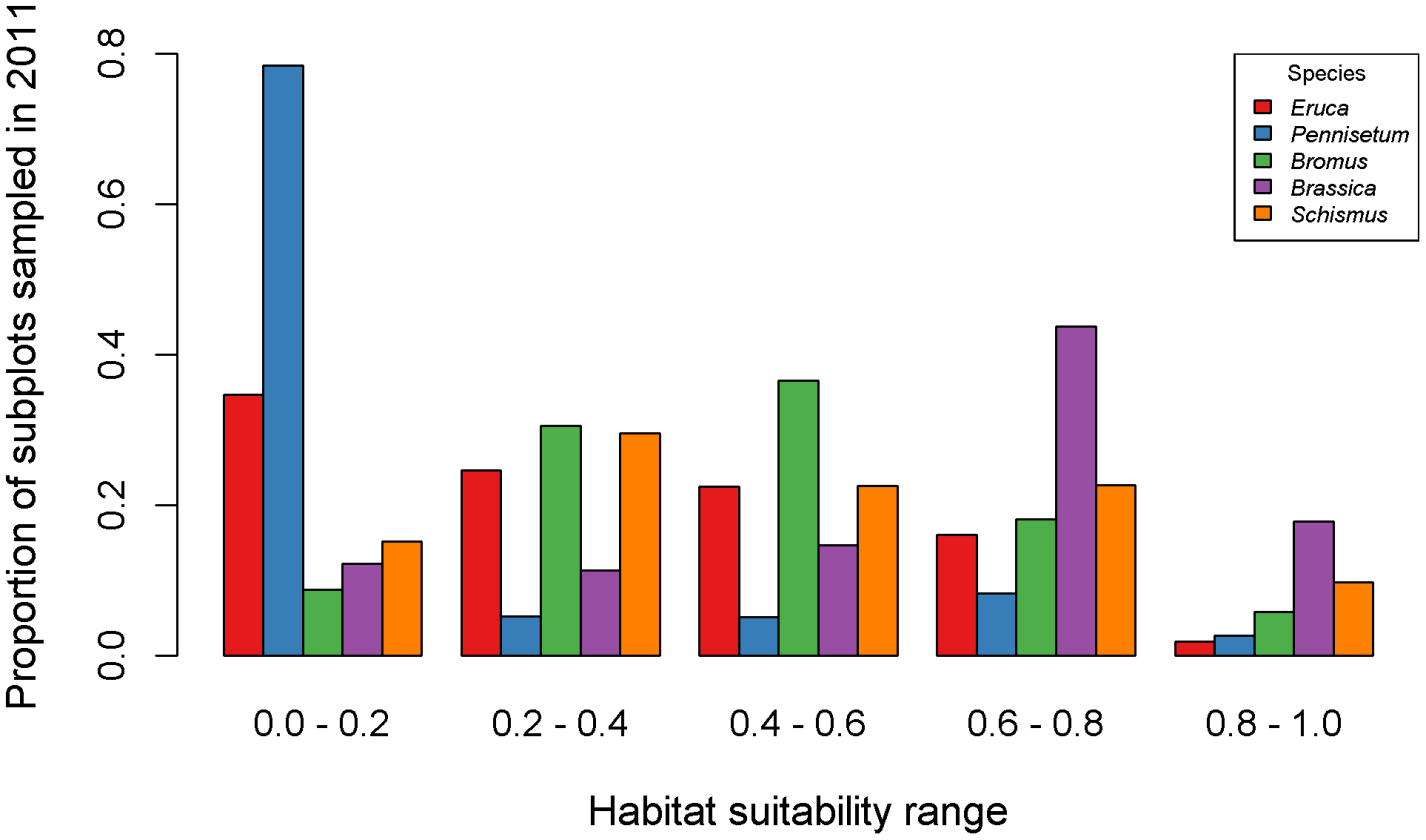
Proportion of sampled subplots in 2011 across habitat suitability ranges for each species. X-axis shows average habitat suitability predicted by five models for each focal species. Y-axis indicates the proportion of subplots that fell within a given range of predicted habitat suitability. We sampled all focal species in habitats that ranged from low to very high suitability to increase chances of detecting unknown populations or unknown areas of species distribution.

By sampling each focal species in habitats that ranged from low to very high predicted suitability, we retained opportunities of detecting unknown populations or unknown areas of species distribution. For example, approximately 37% of the sampled subplots were within low to medium habitat suitability for *Brassica* (0–0.6 in predicted suitability), thus enabling us to characterize *Brassica* distributions in areas where detections were previously unrecorded (Figure 3). Similarly, we sampled at subplots across a wide range of habitat suitability for *Schismus* (0–1 in predicted suitability), allowing us to sample populations located in areas not known to be occupied by *Schismus* (Figure 3). We sampled at fewer subplots predicted with medium to high habitat suitability for *Pennisetum*, as a result of prioritizing sampling efforts in hot desert ecosystems with sandy soils where *Pennisetum* was less common.

### Detections in 2011 and correspondence between detections and habitat suitability models

We detected presence of at least one of our five focal species at 184 (77%) plots and 686 (59%) subplots. We detected *Schismus* more frequently than the other four species, with a 56% detection rate in 2011 at plots and 43% at subplots. *Brassica* was less common, but was still detected in 47% of the plots and 28% of the subplots (Table 3). *Brassica* exhibited considerable clustering, with relatively higher abundance on subplots prioritized for measuring sandy sites across the study area. It was also locally more abundant than *Schismus* even though it was less frequently detected. *Bromus*, *Eruca*, and *Pennisetum* were relatively uncommon. Locations where we detected *Bromus* and *Pennisetum* appeared to be at the edge of their predicted suitable habitat, whereas *Eruca* occurred in few clustered populations in highly localized areas. Individual plants were small in stature and cover was sparse, as all five focal species showed an average percent cover <1% based on point intercept counts along transects in each subplot. At some xero-riparian locations, we observed that plants sampled grew beneath desiccated focal species from the previous year's production that had attained a much greater size (e.g. *Brassica*) and cover during a relatively wet year.

**Table 3:** Number and percentage of detections of five focal species by plot and subplot sampled in the Sonoran Desert of Arizona during our 2011–2012 field seasons.

	2011 Detections		2012 Detections	
Species	Plot (n = 238)	Subplot (n = 1,171)	Plot (n = 506)	Subplot (n = 2,530)
*Schismus*	133 (56%)	505 (43%)	473 (93%)	2020 (80%)
*Brassica*	113 (47%)	329 (28%)	260 (51%)	748 (30%)
*Bromus*	15 (6%)	54 (5%)	11 (2%)	13 (0.5%)
*Eruca*	14 (6%)	32 (3%)	26 (5%)	77 (3%)
*Pennisetum*	21 (9%)	46 (4%)	3 (0.6%)	3 (0.1%)

**Figure 4:**
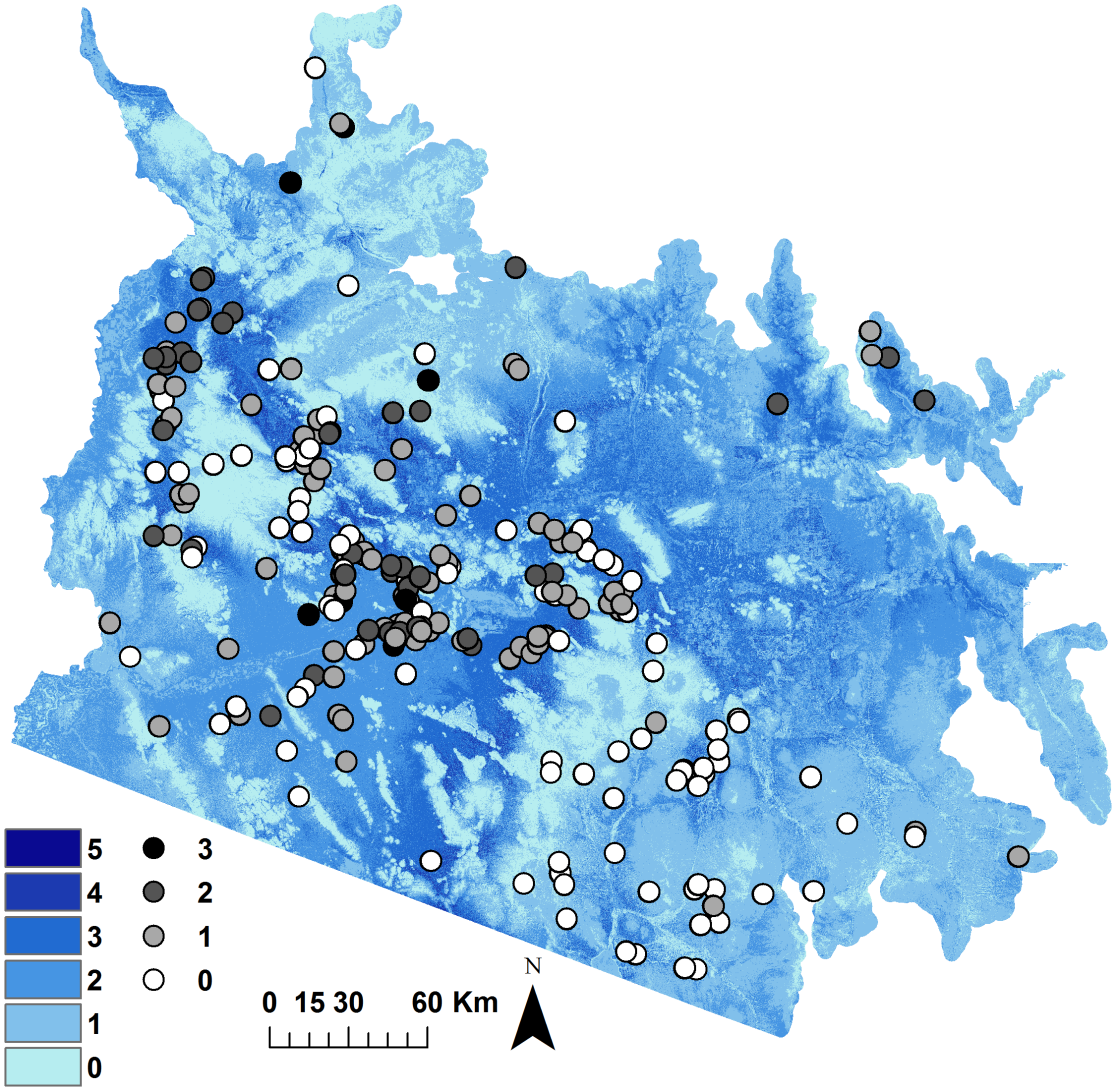
Number of species (black, gray, and white circles) detected in our study area in 2011. Colored areas show the number of habitat suitability models (Model 4 for winter annuals and Model 5 for *Pennisetum*) with predicted high habitat suitability (70^th^ percentile). Darker colors indicate greater spatial overlap of high suitability across species.

We found high correspondence between locations where our focal species were detected and areas of high habitat suitability. Most of our detections fell within the 70^th^ percentile of Model 4 for winter annuals and Model 5 for *Pennisetum* (Figure 4). Among the 686 subplots with presence of at least one out of five focal species, 652 (95%) fell within the 70^th^ percentile of at least one of these HSMs (Figure 4). Furthermore, 206 (80%) of the 257 subplots that had presence of multiple (n≥2) focal species were within the 70^th^ percentile of more than one of these HSMs (Figure 4). More specifically, for the two most common species at the plot level, *Schismus* and/or *Brassica* was present in 70% of the sampled locations, and 92% of these plots corresponded to the 70^th^ percentile of Model 4 for either or both species. Similarly, we detected *Schismus* and/or *Brassica* in 54% of the sampled subplots, among which 89% fell within the 70^th^ percentile of Model 4 for either or both species. Greater variation in habitat suitability occurred in plots/subplots where *Schismus* was present. At locations with presence of *Schismus*, only 54% at plots and 49% at subplots corresponded to the 70^th^ percentile of *Schismus* HSM. In contrast, 90% of the plots and 87% of the subplots where *Brassica* occurred were within the 70^th^ percentile of *Brassica* HSM.

**Figure 5:**
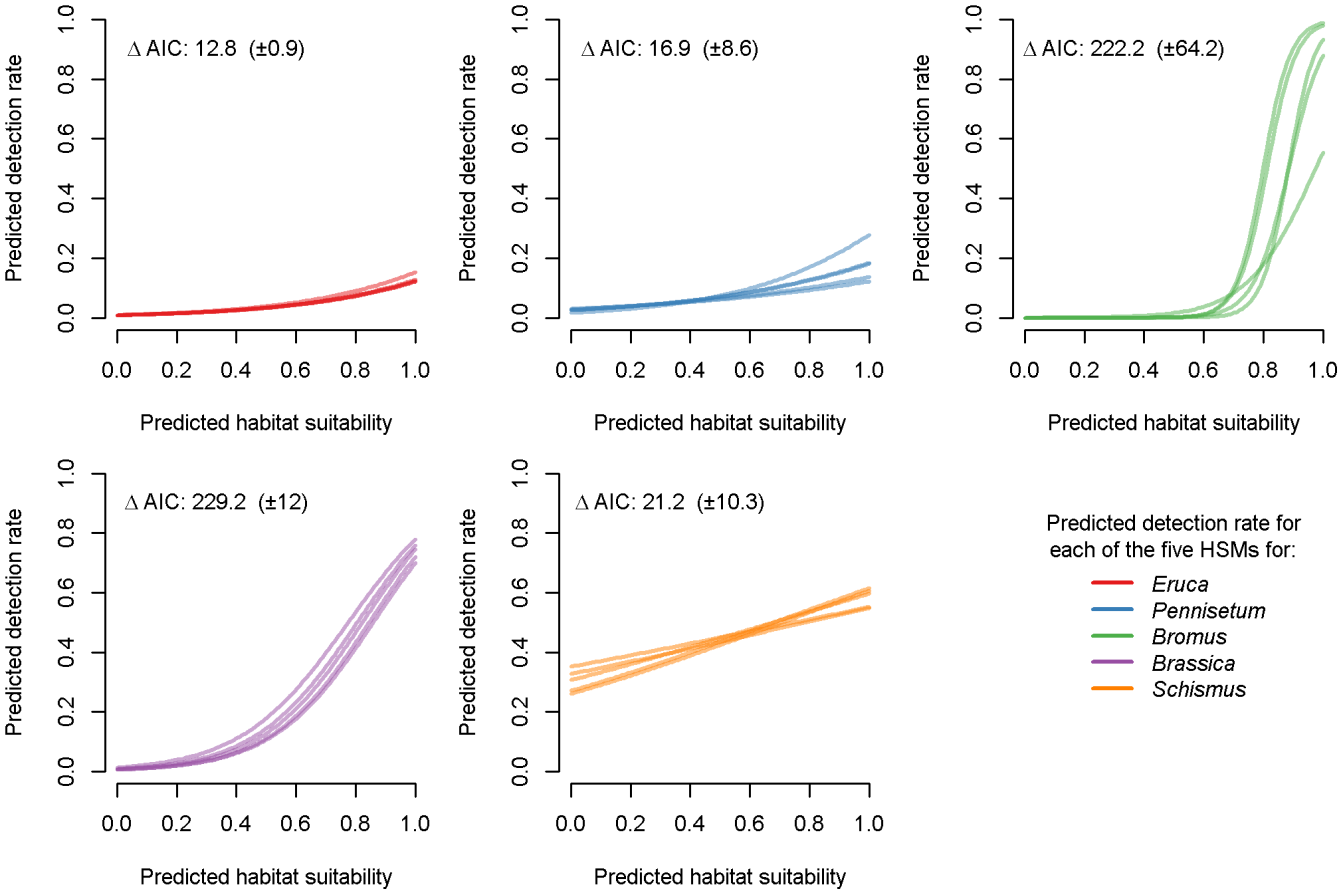
Relationship between predicted habitat suitability and modeled detection rate at subplots for each of the five habitat suitability models for each focal species. We used a generalized linear model to fit regression line between binary field detections in 2011 and predicted habitat suitability. Detections were modeled using a binomial distribution and a logit link function. For each focal species, we show the average delta Akaike Information Criterion (ΔAIC) ±95% confidence interval for models of detection rate that included predicted habitat suitability versus models that included an intercept term only.

Modeled detection rates for each focal species varied over the range of habitat suitability with mixed relationships. Corresponding to our field detections, *Brassica* models performed the best, showing the strongest positive relationship between predicted detection rates and habitat suitability. All five *Brassica* models showed a positive trend of increasing detection with higher suitability (e.g. predicted detection rate was >0.7 when habitat suitability was >0.8) (Figure 5). Four of the five *Bromus* models showed high predicted detection rate (>0.8) at high habitat suitability (>0.8) and all five models predicted nearly non-detections at low to medium habitat suitability, corresponding to our finding of abundant *Bromus* populations at few locations (Figure 5). Corresponding to the widespread field detections of *Schismus* across different habitats, all five *Schismus* models showed positive but less strong relationships, as predicted detection rates ranged from low to medium (0.2–0.6) across low to very high habitat suitability (Figure 5). Predicted detection rates were low (less or near 0.2) across ranges of habitat suitability for *Eruca* and *Pennisetum* models, reflecting our finding that the populations were regionally rare and locally abundant at only a few locations in the study area (Figure 5). All 25 predictive models of detection rates outperformed models that only included regression intercepts when predicted habitat suitability was included in the models. The average ΔAIC was >10 (i.e. our threshold of model goodness of fit) for all five models for each focal species (average ΔAIC  = 12.8–229.2) (Figure 5). *Brassica* and *Bromus* models showed the strongest fit, whereas *Eruca* and *Pennisetum* models were the weakest.

### Improved sampling efficiency and detections in 2012

Despite a second year of below average rainfall following 2011, both sample size and detection rates in 2012 increased greatly with iterative adjustments by integrating additional HSM input data, stratification of HSMs and other vegetation indices, and more rigorous sampling location prioritization and targeting. We sampled 10 and 50 plots and subplots per week in 2011 versus 18 and 92 plots and subplots per week in 2012 (i.e. 506 plots and 2,530 subplots were sampled in 2012). The overall detection of at least one of our focal species increased from 77% in 2011 to 96% in 2012 at plots and from 59% in 2011 to 84% in 2012 at subplots. In particular, the detection of *Schismus* nearly doubled and reached 93% at plots and 80% at subplots (Table 3). All focal species had sparse populations, with an average percent cover <3% at subplots because of low overall annual plant productivity for the study area in 2011 and 2012. Among the subplots where we detected our focal species, 93% fell within the 70^th^ percentile of at least one of species HSMs. More specifically, 76–78% of the detections for *Schismus* and *Brassica* at plots and subplots occurred within the 70^th^ percentile of species HSMs. The correspondence between detections and ratio of 2001–2010 maximum spring NDVI to mean spring NDVI was slightly lower. We found that 65–68% of overall detection for at least one focal species at plots and subplots fell within the 60^th^ percentile of this index of vegetation productivity.

## Discussion

### The environmental characteristics of invasion

Our Model 4 for winter annuals and Model 5 for *Pennisetum* indicated that 81% of the study area was predicted with high (i.e. 70^th^ percentile) habitat suitability for at least one of the focal species. Furthermore, 42% of the study area had highly suitable habitats for multiple (≥2) species. The wide spatial distribution of areas considered suitable habitat suggested that the extent to which potential invasion could occur is larger than the current species distribution, particularly for years experiencing above average rainfall. Because we sampled across habitat suitability gradients for each species, across environmental gradients, and away from easily accessible roads, we were able to obtain a more realistic estimate of the extent of invasion across the study area despite the two relatively dry winter seasons that preceded sampling.

*Brassica* appeared to dominate extremely sandy soils and dunes [32], [43], whereas *Schismus* was more generally distributed, occurring ubiquitously as a minor component of most plant communities in the lower Colorado River desert. Given the relatively recent introduction of *Eruca*, its true fundamental niche may not be well characterized by HSMs. *Eruca* may be in the early stages of expansion and can likely cover a much more extensive range than it currently inhabits [66]. On the other hand, *Bromus* and *Pennisetum* appear better suited to Sonoran Desert uplands with higher winter and summer precipitation than what is typical of the lower Colorado River subdivision [27]. Our results suggested that *Bromus* and *Pennisetum* are likely to remain rare in the hottest desert areas of the Sonoran Desert with extremely low summer rainfall (Table 1). In comparison, during our preliminary sampling in 2010 at locations with great abundance of *Pennisetum* in south-central Arizona where the mean annual precipitation is 32.3 cm [35], we detected much higher presence (47%) and cover (11%) (Wang et al., unpublished data). Sánchez-Flores found that HSMs derived from anthropogenic variables showed relatively greater predictive power than environmental (non-climatic) variables for *Brassica* and *Schismus* [43]. In our study, however, we did not find evidence that disturbance factors, such as distance to the nearest road, was a stronger predictor than the environmental variables (climatic and biophysical) we considered. Our finding suggested that combining climatic, biophysical, and disturbance factors can improve prediction performance and better characterize the spatial extent of plant invasions that are likely to have a broader distribution than is currently recognized without large-scale multi-jurisdictional surveys [43], [67].

### Detection rates across ranges of habitat suitability

Our HSMs and field detection results indicated that a model-informed, iterative and targeted sampling design not only characterized important environmental attributes, but also improved the detectability of some focal species. By sampling across habitat suitability ranges, the detection rates in 2011 for *Schismus* at plot and subplot levels and *Brassica* at plot level outperformed all detection rates by another multi-species study that used simulated field detections and multiple field sampling methods [10]. Furthermore, by employing a rigorous targeted approach to devising the sampling framework used in 2012, we greatly increased the sample size and number of detections for some focal species, especially *Schismus*. In addition, this sampling approach yielded an opportunity to evaluate how well HSM-informed stratification corresponded to species detections. Overall, modeled detection rates showed notable variation in correspondence with HSM predictions among the five species studied. Observations of high correspondence between high habitat suitability and increasing detection rates for *Brassica* supported the use of HSMs to detect populations and the potential spatial extent of invasion. There are many suitable locations that *Brassica* could colonize but has not yet reached, or where it was once abundant but has decreased during consecutive years of low rainfall. *Eruca* is less well distributed, as evidenced by its highly localized and overall low detections in areas predominated by high habitat suitability. This species appears to be dispersed along recently abandoned agricultural fields and washes, but it also has the potential to occupy relatively undisturbed locations, such as sandy areas with surrounding rocky terrain. The detection rate for *Schismus* was less variable across the range of habitat suitability, perhaps because this species occupies a broad range of environmental conditions.

### Other factors that influenced field detections

Some limitations existed despite a high level of detection for certain species achieved by our sampling design. First, we constructed HSMs by leveraging available datasets that represented variable sampling efforts and might not fully represent the distribution of a given species in the study area. Both *Schismus* and *Eruca* could be missed from roadside sampling because small *Schismus* plants are often quite small in stature and less easily identified, and *Eruca* is less widespread. In contrast, *Brassica* is better documented because of its large and conspicuous form especially during wet years [32], [43]. We attempted to reduce bias by sampling reasonable distances away from roadsides and across large environmental gradients (i.e. habitat suitability ranges) to capture variation that opportunistic sampling would not have been able to accomplish. To treat such non-detection sampling bias, the next iteration of HSMs could incorporate recommendation from Hefley et al. to elicit experts to provide auxiliary data for estimating the probability of detection [15]. Second, our HSMs provided landscape- to regional-scale habitat suitability information to stratify potential occurrence locations and identify uninhabited areas that are suitable for colonization [68]. Therefore, the landscape to regional trend of suitable habitats predicted by HSMs may not capture the local- or fine-scale variation of colonized habitats. Third, HSMs based on presence-only data do not imply species presence or absence because species could be absent from highly suitable habitats or present in less suitable habitats due to biotic, historical, or dispersal factors [69]–[71]. For example, *Eruca* currently occupies a very low proportion of its predicted suitable habitats, but may have great capacity to expand its distribution during years of increased plant productivity (e.g. El Niño events).

Fourth, our HSMs did not include process-based models to couple predictions with dispersal dynamics and empirically examine the effects of invasion stages on the degree of non-equilibrium in models (e.g. [72]). We attempted to capture these types of non-stationary factors by: 1) sampling across large environmental gradients to capture a wide range of conditions that could support species dispersal, colonization, and establishment, and 2) sampling during two years and spring growing periods to capture the dynamic process of dispersal, colonization, and establishment that might have occurred in one year, but not the other as limited rainfall in the study area was highly and spatially heterogeneous among years. Refining future sampling efforts could implement dispersal constraints into habitat suitability models by quantifying the probability of dispersal as a function of distance from the source population (i.e. location with presence records) [73], [74]. To overcome the challenge of deficiency in empirical data required for model parameterization (e.g. [75]–[77]), we could implement the simplest dispersal characteristics (e.g. distance to the nearest source population for each pixel) and then employ a more rigorous targeted strategy that considers the distribution of locations within the neighborhood of invasion hotspots identified from HSMs, expert knowledge, and previous sampling efforts. For example, we could focus on areas with recent fires or substrates that would facilitate the dispersal and establishment of our focal species.

Finally, the low winter precipitation prior to sampling affected our overall detection rates. Indeed, total precipitation in Yuma County from December, 2010 to April, 2011 was 37% below the previous ten year average (2000–2010) and 76% below the wettest year on record (2005) (Western Regional Climate Center 2011; http://www.wrcc.dri.edu/). Detection rates and percent cover were lower than what might be expected during a wetter year, therefore, focal species were absent or sparse from areas that would ordinarily have invasive plants present or with greater cover. We observed that most of our focal species tended to occupy only shaded areas beneath native shrub canopies, within drainages or at roadsides which have pseudo-riparian characteristics such as higher soil moisture and fertilization effects [32].

### Recommendations to managers

Our iterative and targeted sampling design allowed us to improve detections for sparse and patchy populations by stratifying locations using HSMs and other ancillary data important to focal species establishment. Our framework is particularly important to fragile ecosystems where varied rainfall patterns may facilitate periodic and large increases in non-native invasive plant production and fuel loads followed by dry periods of increased fire risk. We demonstrate how modeling results can be used to guide the design of management protocols by explicitly linking model-informed sampling to management strategies [56]. Accordingly, we identified strategies for improving detections rates that are also applicable to other species and ecosystems. First, location selection using strata predicted by HSMs should include a greater range of habitat suitability that covers more local habitat variation. Extending the HSM approach and adding other stratified vegetation indices such as remote sensing-derived phenological metrics in vegetation greenness can facilitate locating areas of greater focal species productivity and abundance. Second, implementing a stratified random and spatially confined approach using rigorous criteria, can increase sample size and further reduce transportation and labor costs. Third, plot prioritization for sampling can be based on the most recent vegetation indices from remotely sensed imagery. This information helps capture vegetation greenness by annual plants prior to field work and helps to avoid sampling in areas with low or no annual plant production. Finally, for focal species that show strong habitat preferences to particular substrate types, incorporating soil substrate maps derived from spectral mixture analysis of high resolution field spectrometer data and satellite image classification can guide sampling prioritization. Spectral end members from other common substrates will also aid avoiding locations that have low potential to support focal species establishment.

Our iterative and targeted sampling design and HSMs provide practical use of existing invasive plan distribution data and useful utility for developing sampling strata and detecting focal species over large geographic areas to satisfy key management objectives: 1) detecting populations of non-native invasive plants across previously unsampled gradients, and 2) characterizing the distribution of non-native invasive plants at landscape to regional spatial scales. We have rigorously examined the iterative and targeted sampling design in a landscape where species invasions pose a threat to native plant composition and structure that are likely to undergo community shifts in the coming decades as a result of climate change. Climate change may enhance processes from introduction to spread of invasion by increasing the transport of propagules, decreasing the resistance of native species to invasion, reducing the space suitable for native species, and creating shifts in ecosystem distributions [78], [79]. Thus, our sampling design framework can play a key role in facilitating monitoring and mitigation activities by land management agencies. Moreover, our novel approach to the nested integration of common and freely available satellite images with field data can be readily extended to other species and ecosystems. Our results highlighted where potentially suitable habitats might be vulnerable to invasion by one or more of our focal species and where monitoring efforts might be focused. Importantly, our methods and results provide a framework for establishing an “early warning system” that is critical to helping managers to recognize the possible extent of future problematic non-native invasive plants across multiple land ownerships.
